# A RIFLE score-based trigger for renal replacement therapy and survival after cardiac surgery

**DOI:** 10.1186/cc10953

**Published:** 2012-03-20

**Authors:** A Schneider, G Eastwood, S Seevanayagam, G Matalanis, R Bellomo

**Affiliations:** 1 Austin Health, Heidelberg, Australia

## Introduction

It is controversial whether all critically ill patients with RIFLE-F class acute kidney injury (AKI) should receive renal replacement therapy (RRT). We reviewed the outcome of open-heart surgery patients with severe AKI who did not receive RRT.

## Methods

We identified all patients who developed AKI after cardiac surgery during a 4-year period, and obtained baseline characteristics, intraoperative details and in-hospital outcomes. We analyzed physiological and biochemical features at the time of RRT initiation or at peak creatinine if no RRT was provided.

## Results

**Figure 1:**
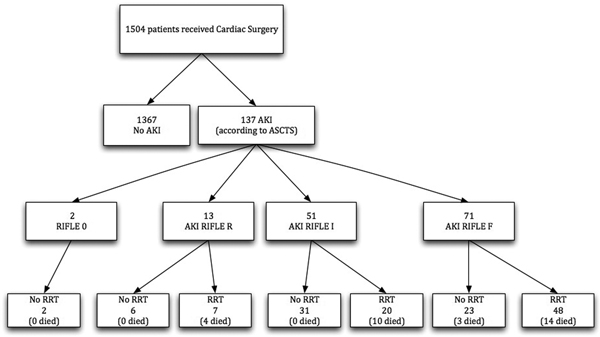
**Flow chart**. ASCTS, Australian Society of Cardio Thoracic Sugery.

We reviewed 1,504 patients. Of these, 137 (9.1%) developed postoperative AKI with 71 meeting RIFLE-F criteria and 23 (32.4% of RIFLE-F cases) not receiving RRT. Compared with RRT-treated RIFLE-F patients, no-RRT patients had lower APACHE III scores, less intraaortic balloon pump requirements, shorter intensive care stay and a trend toward lower mortality. At peak creatinine, their urinary output, arterial pH and PaO_2_/FIO_2 _ratio were all significantly higher. Their serum creatinine was also higher (304 vs. 262 μmol/l, *P *= 0.02). Only three died in-hospital. Detailed review of cause and mode of death was consistent with non-RRT-preventable deaths. In contrast, 27 patients with RIFLE-R or RIFLE-I class received RRT. Compared with RRT-treated RIFLE-F patients, they had a trend towards a more severe presentation and a higher mortality (51.8% vs. 29.2%, *P *= 0.02). See Figure 1.

## Conclusion

After cardiac surgery, RRT is typically applied to patients with the most severe clinical presentation irrespective of creatinine levels. A RIFLE score-based trigger for RRT is unlikely to improve patient survival.

